# Feeding experiments on *Vittina turrita* (Mollusca, Gastropoda, Neritidae) reveal tooth contact areas and bent radular shape during foraging

**DOI:** 10.1038/s41598-021-88953-7

**Published:** 2021-05-05

**Authors:** Wencke Krings, Christine Hempel, Lisa Siemers, Marco T. Neiber, Stanislav N. Gorb

**Affiliations:** 1 Department of Mammalogy and Paleoanthropology, Center of Natural History (CeNak), Universität Hamburg, Martin-Luther-King-Platz 3, 20146 Hamburg, Germany; 2 Department of Functional Morphology and Biomechanics, Zoological Institute, Christian-Albrechts-Universität zu Kiel, Am Botanischen Garten 9, 24118 Kiel, Germany; 3 Department of Animal Diversity, Center of Natural History (CeNak), Universität Hamburg, Martin-Luther-King-Platz 3, 20146 Hamburg, Germany

**Keywords:** Zoology, Biomechanics, Structural biology

## Abstract

The radula is the food gathering and processing structure and one important autapomorphy of the Mollusca. It is composed of a chitinous membrane with small, embedded teeth representing the interface between the organism and its ingesta. In the past, various approaches aimed at connecting the tooth morphologies, which can be highly distinct even within single radulae, to their functionality. However, conclusions from the literature were mainly drawn from analyzing mounted radulae, even though the configuration of the radula during foraging is not necessarily the same as in mounted specimens. Thus, the truly interacting radular parts and teeth, including 3D architecture of this complex structure during foraging were not previously determined. Here we present an experimental approach on individuals of *Vittina turrita* (Neritidae, Gastropoda), which were fed with algae paste attached to different sandpaper types. By comparing these radulae to radulae from control group, sandpaper-induced tooth wear patterns were identified and both area and volume loss could be quantified. In addition to the exact contact area of each tooth, conclusions about the 3D position of teeth and radular bending during feeding motion could be drawn. Furthermore, hypotheses about specific tooth functions could be put forward. These feeding experiments under controlled conditions were introduced for stylommatophoran gastropods with isodont radulae and are now applied to heterodont and complex radulae, which may provide a good basis for future studies on radula functional morphology.

## Introduction

Gastropod diversity is accompanied by feeding on a variety of ingesta sources allowing the establishment of ecological niches. The interface between animal and food intaken is the radula consisting of a thin, flexible, and chitinous membrane with teeth arranged in transversal and longitudinal rows^1,2^. Both are constantly secreted in radulas’ posterior part, the building zone, and digested in its anterior part, the degenerative zone^2–10^. During foraging, this membrane is either pulled or spanned between odontophoral cartilages by buccal mass musculature resulting in tearing of ingesta and collection of loosened particles. Sliding contacts and interactions between teeth and substrate or food eventually lead to tooth wear.

As quantity, shape, arrangement, material properties, and chemical composition of teeth can be highly distinct, within one individual radula and between molluscan taxa, hypotheses about functional specializations of tooth types are at hand. Additionally, many hypotheses about radular adaptation to food-types had been put forward. Past approaches on radular functional principles involve observation and documentation of foraging behavior^11–16^ or analysis of radular feeding tracks^2,11,12,15,17–24^. However, as radular motion is highly complex, teeth are rather small, and feeding tracks are difficult to interpret, precise function and interplay between different radular structures (membrane, tooth rows, odontophoral cartilages, and buccal mass musculature) still await further structural and especially experimental investigation. Additionally, to deeply understand the functionality of a grinding tool—here the radular tooth—, its precise area of contact—here with ingesta (substrate, food, everything that is taken in)—must be determined. This is essential from a tribological point of view, since the morphology of the interacting part determines its capability to transfer forces (^25–33^; for review see^34^). These analyses might also contribute to the development of radula-inspired, soft grasping robotic applications, as introduced on *Aplysia* (Heterobranchia; radula with small, isodont teeth)^35^.

To determine tooth contact areas and to reconstruct the interplay between radular structures, we here present an approach involving feeding experiments with the neritid gastropod *Vittina turrita* (Gmelin, 1791^36^) on sandpapers of distinct dimension of particles and therefore with different abrasiveness. These experiments under controlled conditions were established for stylommatophoran gastropods with isodont teeth^37^ and are now transferred to complex and heterodont radulae. Our approach led to the determination of contact areas and of the radular configuration during foraging.

## Methods

### Animals studied

For feeding experiments, 40 individuals of *Vittina turrita* (Gastropoda: Neritidae) were obtained from an online pet shop (pet shop is online available at *garnelio.de*, here gastropods are sold as *Neritina turrita*). This species was chosen for our experiments, because (1) it possesses a heterodont radula with very distinct tooth morphologies and sizes, (2) animals are easy to obtain, shelter, and feed, (3) and videos of radular motion are available^16^.

**Figure 1:**
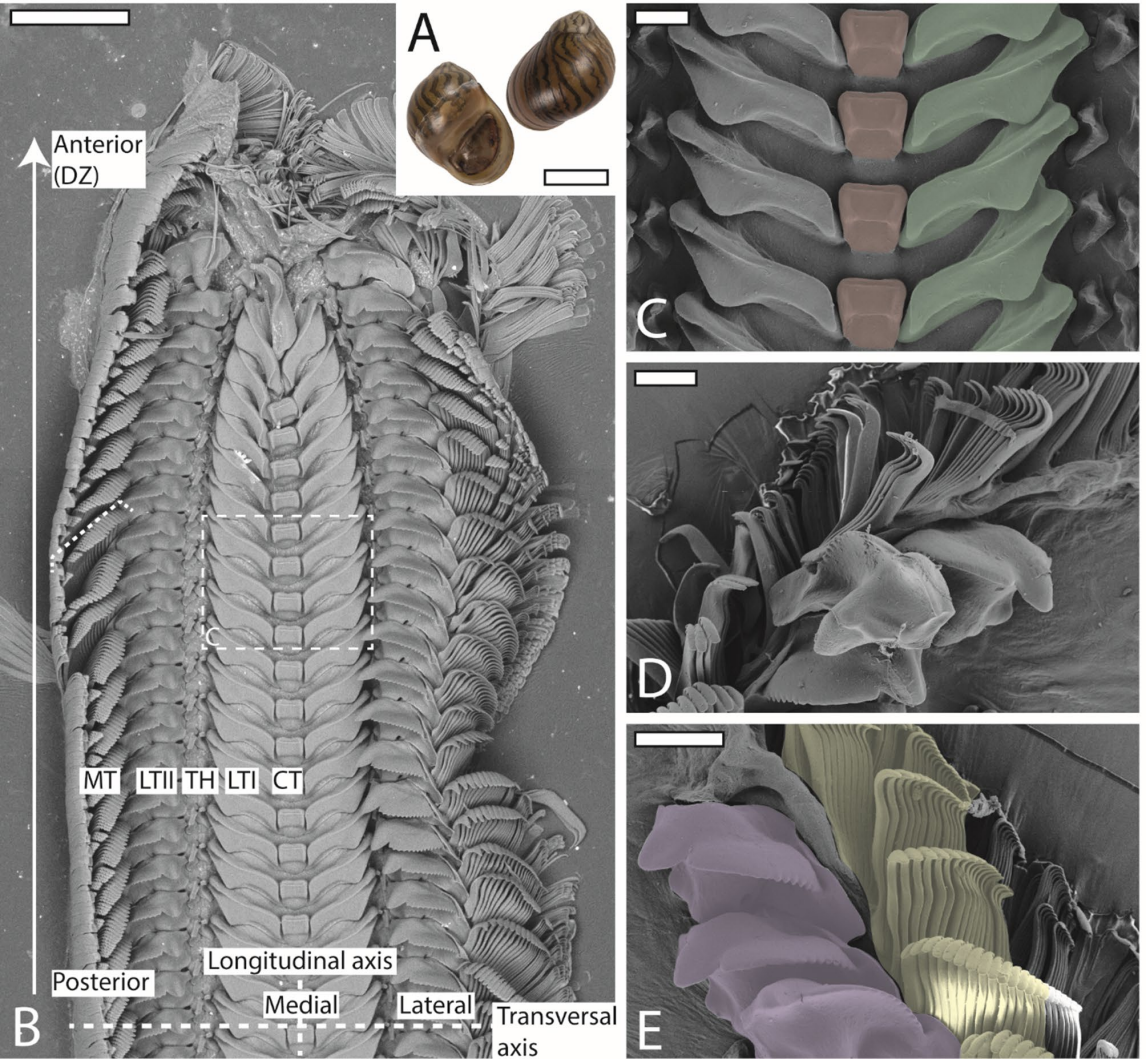
(**A**) Shell of *Vittina turrita*. (**B**–**E**) SEM images of one radula from control group. B. Image of outermost part of radula (wear and digestive zone; arrow points in direction of increasing wear) with definition of the anatomical directions (medial, lateral, anterior, posterior) and radular axes (transversal and longitudinal axis). (**C**) Central teeth (red) and lateral teeth I (green). (**D**–**E**) Lateral tooth II (purple) and inner 15 marginal teeth (yellow) of degenerative zone. CT = central tooth, DZ = degenerative zone, LTI = lateral tooth I, LTII = lateral tooth II, MT = marginal teeth, TH = thickening of membrane/interlocking teeth. Scale bars: A = 30 mm, B = 250 μm, C–E: 60 μm.

*Vittina turrita*, with a shell height of 15–26 mm (Fig. 1A), inhabits brackish mangroves and riverine environments between Indonesia and Thailand^38^. Since these gastropods need brackish water to breed, *V. turrita* is usually collected in its habitat and exported for international aquarium trade. Species determination was conducted after experiments on the basis of shell and operculum morphology following^38^.

### Experimental set-up

**Figure 2:**
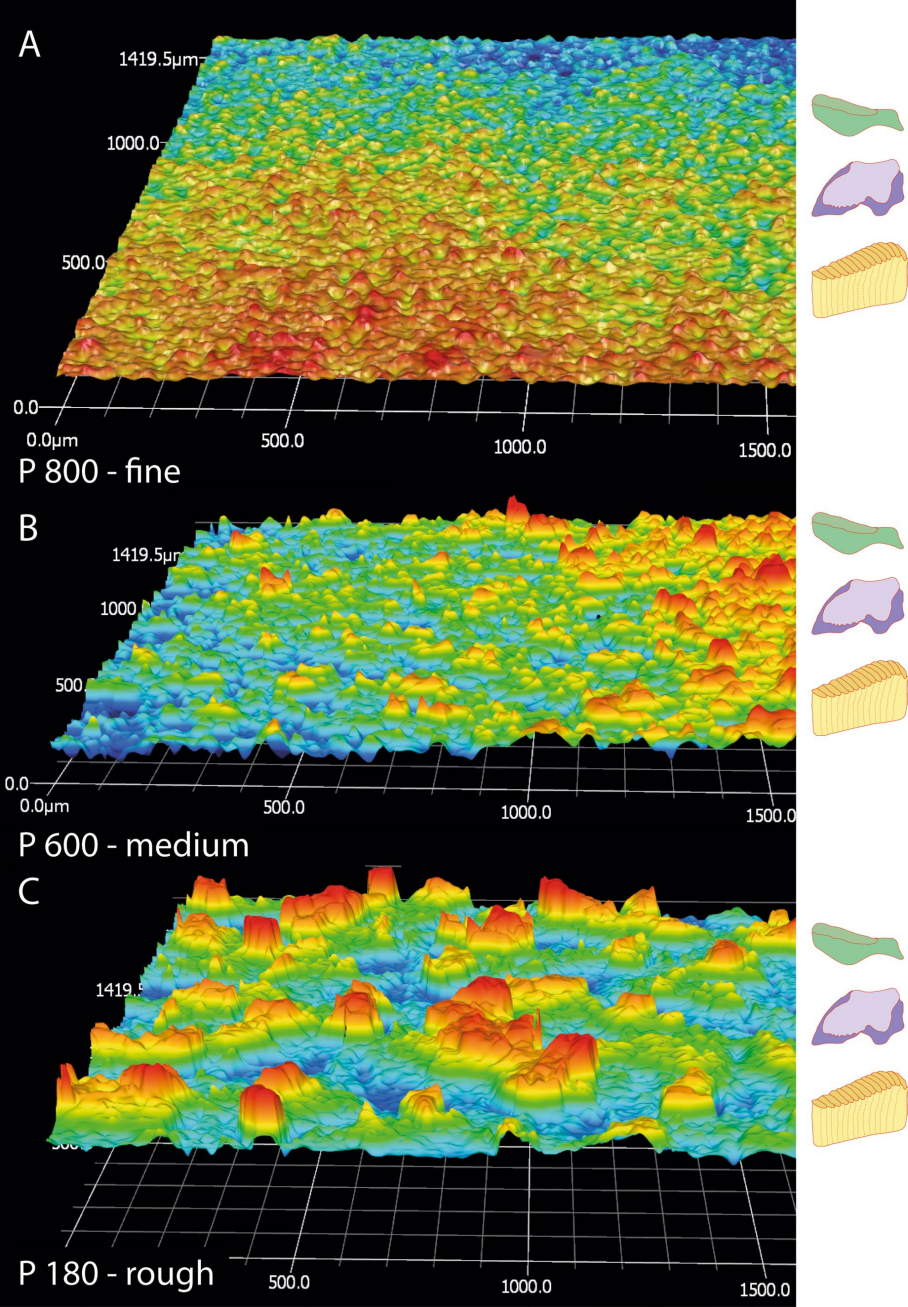
Relative dimensions of sandpaper profiles used in experiments and schemes of radular teeth at same scale. Left side: Surface profile of sandpapers visualized with the Keyence VR 3100. (**A**) = fine (P 800), (**B**) = medium (P 600), (**C**) = rough (P 180) sandpaper; scale bars are in µm. Right side: schematic illustration of the unworn teeth (lateral I, lateral II, inner marginals) drawn with Adobe Illustrator CS 6.

For experiments, adult gastropods were separated into four freshwater aquariums either lined with water-resistant sandpapers (45PCS sandpaper, Shenzhen Take Tools Co., Ltd., Guangdong, China) coated with sharp aluminum-oxide grains of distinct abrasiveness (from fine to rough: P 800 = fine [with N = 10 gastropods], P 600 = medium [with N = 10 gastropods], P 180 = rough [with N = 10 gastropods]) or without sandpaper (control group; N = 10 gastropods). Sandpapers were characterized according to ISO 25,178 employing a Keyence VR 3100 (KEYENCE, Neu-Isenburg, Germany) and quantified (Table 1, Fig. 2), to relate size of sandpaper particles to size of radula and single teeth. For quantification of sandpapers we measured the particles on a straight line of 1500 µm. The parameters given in Table 1 are the mean values. Gastropods were fed daily for seven to eight weeks with algae paste (Schneckenfeed Paste, NatureHolic GmbH, Mannheim, Germany) which was either attached to the glass surface (control group) or to sandpaper. We could not observe gastropods during foraging since a) sandpapers are certainly not transparent and b) Neritidae forage in such a way, that foraging behaviour cannot be observed from the side in contrast to Stylommatophora. Thus, some individuals might have fed more frequently on sandpaper. Additionally, the sandpapers are not homogeneous in their surface structure. However, since clear wear patterns were found (see results) and our aim was the identification of contact areas, this aspect does not weaken the study. After experiments, gastropods were fixed by short boiling, subsequently preserved in 70% ethanol, and inventoried in the malacological collection of the Zoologisches Museum Hamburg (ZMH) of the Centrum für Naturkunde (CeNak) (ZMH 154753).

**Table 1:** Parameters of sandpapers used in feeding experiments (characterized employing Keyence VR 3100).

Grit number	Sandpaper types		
	P 180—rough	P 600—medium	P 800—fine
Roughness, µm, Ra	21.1	5.2	3.8
Mean width, µm, RSm	195.7	28.1	22.3
Mean height of profile elements, µm, Rc	80.6	19.1	17.5

### Documentation of radula and wear analysis: area loss

**Figure 3:**
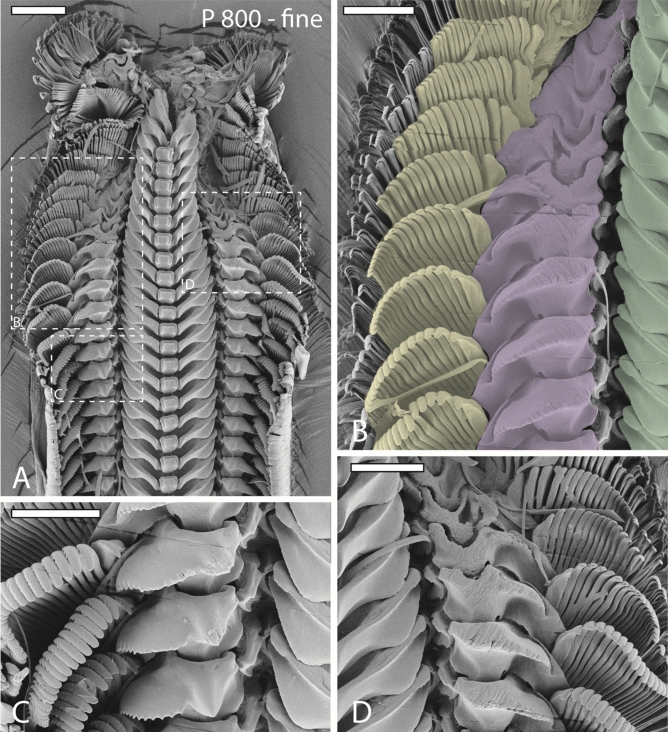
SEM images of one radula from specimen sheltered on fine sandpaper (P 800). (**A**) Image of outermost part of the radula (wear and digestive zone). (**B**–**D**) Magnifications of same region as in (**A**). (**B**) Right wear zone with colored outlines of lateral teeth I (green), lateral teeth II (purple), and inner 15 marginal teeth (yellow; only marginal teeth that show signs of wear were colored). (**C**) First signs of wear on lateral tooth I, lateral tooth II, marginal teeth. (**D**) Left wear zone. Scale bars: A = 200 μm, B–D = 100 μm.

**Figure 4:**
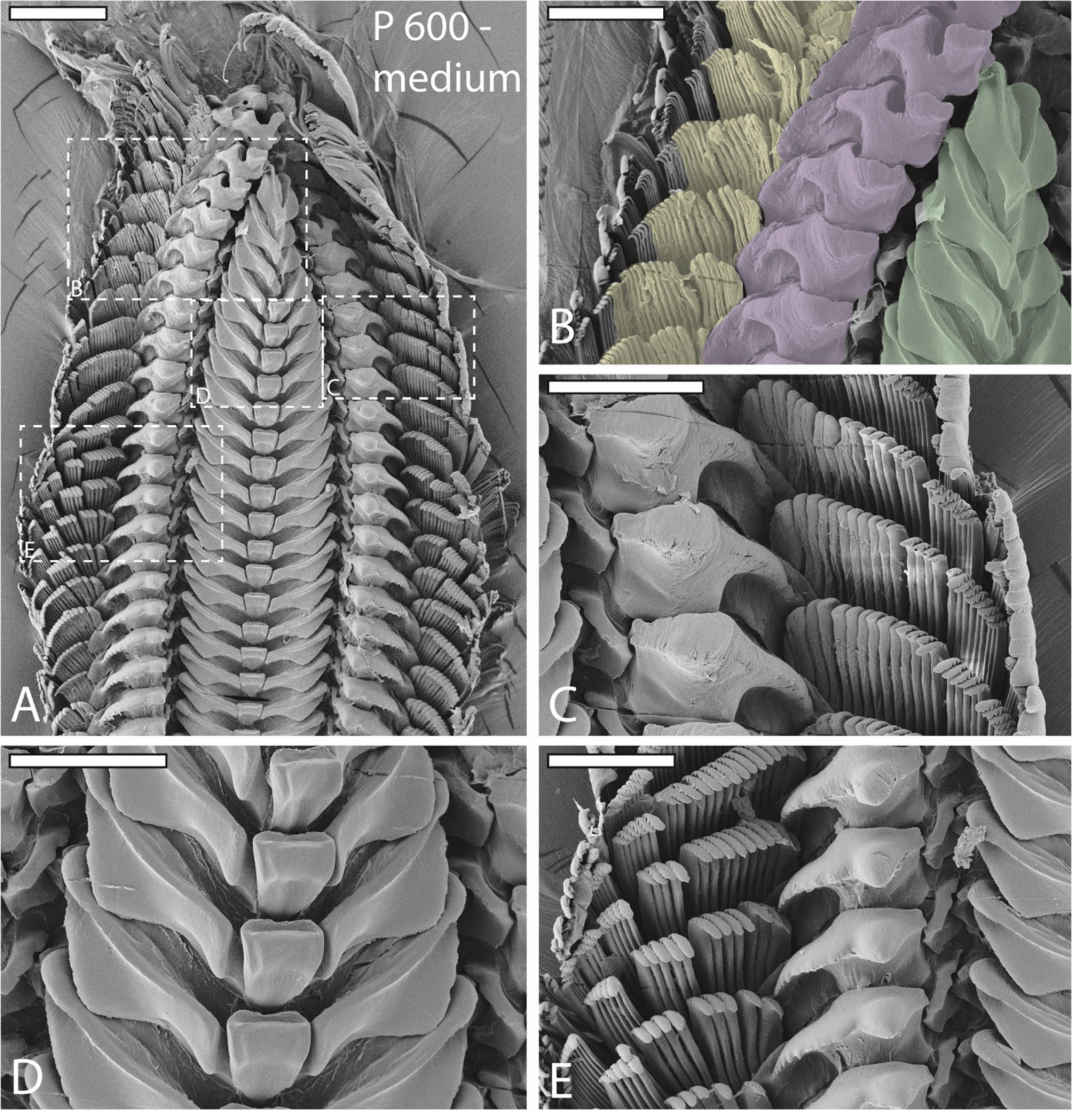
SEM images of one radula from specimen sheltered on medium sandpaper (P 600). (**A**) Image of outermost part of radula (wear and digestive zone). (**B**–**E**) Magnifications of same region as in (**A**). (**B**) Right wear zone with colored outlines of lateral teeth I (green), lateral teeth II (purple), and inner 15 marginal teeth (yellow; only marginal teeth that show signs of wear were colored). (**C**) Left wear zone. (**D**) Worn lateral teeth I and unworn central teeth. (**E**) First signs of wear on lateral teeth I, lateral teeth II, and marginal teeth. Scale bars: A = 200 μm, B–E = 100 μm.

**Figure 5:**
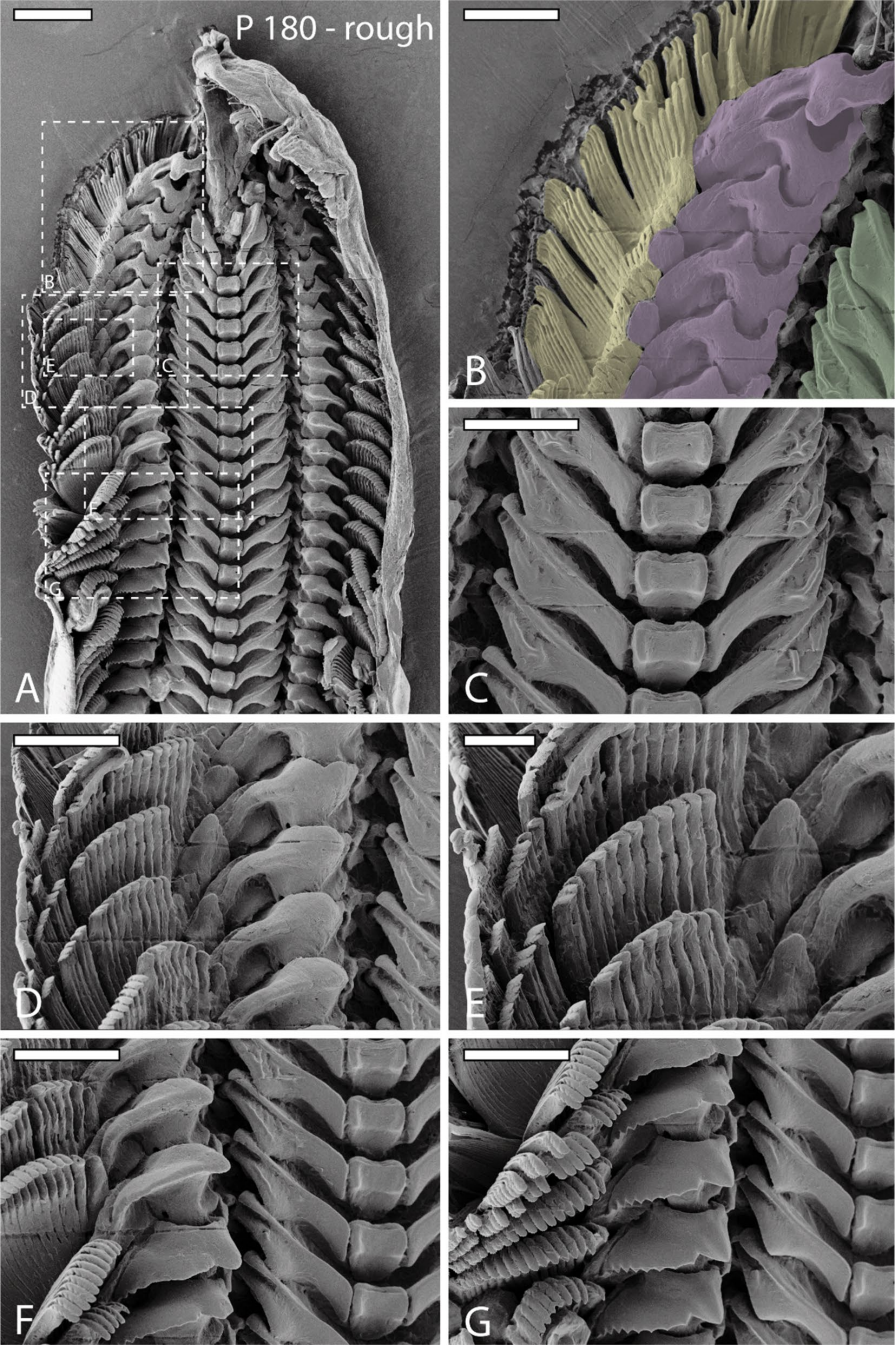
SEM images of one radula from specimen sheltered on rough sandpaper (P 180). (**A**) Image of outermost part of radula (wear and digestive zone). (**B**–**G**) Magnifications of same region as in (**A**). (**B**) Right wear zone with colored outlines of lateral teeth I (green), lateral teeth II (purple), and 15 inner marginal teeth (yellow; only marginal teeth that show signs of wear were colored). (**C**) Worn lateral teeth I and unworn central teeth. (**D**–**E**) Worn lateral teeth I, lateral teeth II, marginal teeth. (**F**) Slight signs of wear. (**G**) First signs of wear on lateral tooth I, lateral tooth II, and marginal teeth. Scale bars: A = 200 μm, B–G = 100 μm.

**Figure 6:**
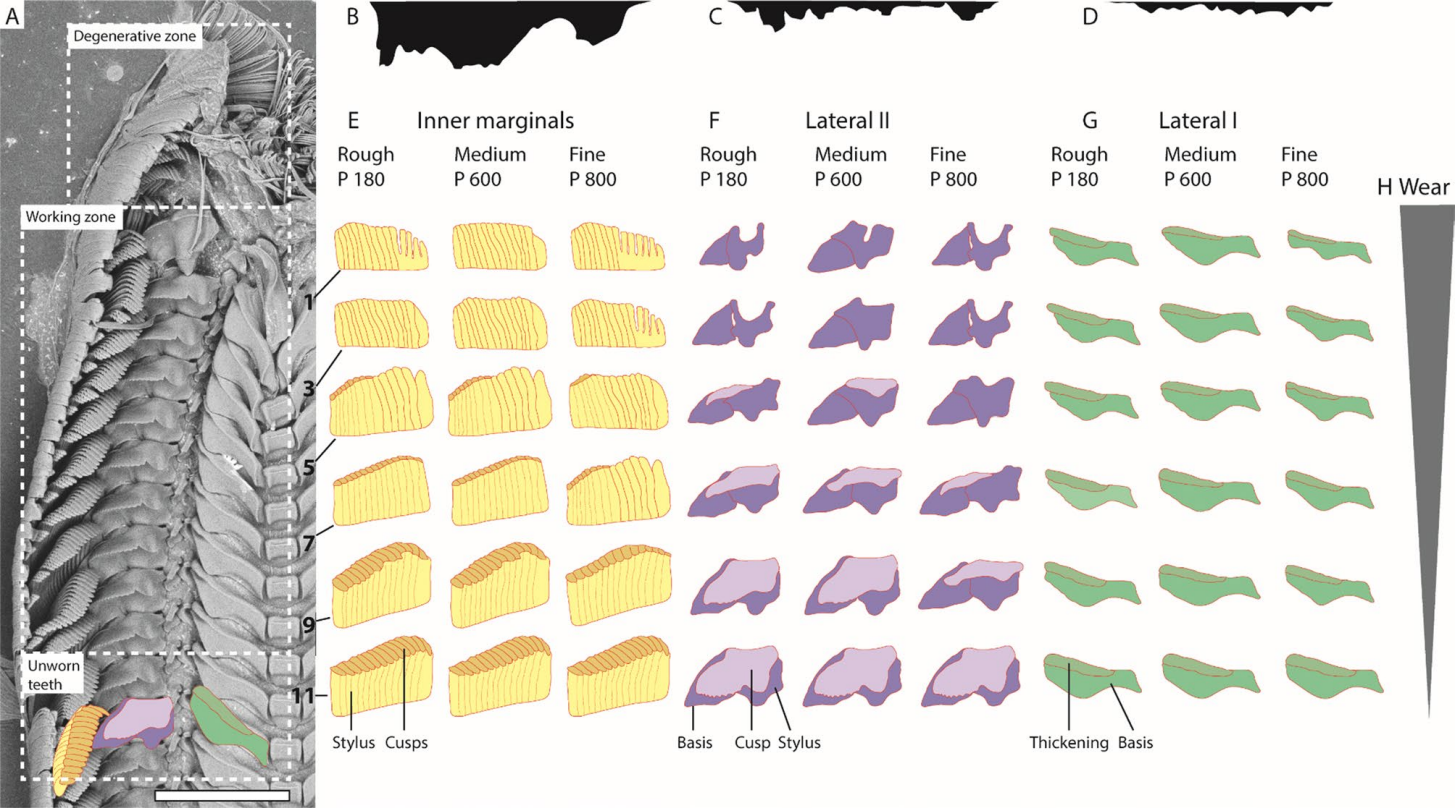
(**A**) SEM images of one radula from specimen of control group, with depicted zones (degenerative zone, working zone, unworn teeth); schematic illustrations of teeth (drawn with Adobe Illustrator CS 6) were plotted on the SEM image to allow identification of tooth schemes (**E**–**G**), scale bar = 200 μm. (**B**–**D**) Schemes (drawn with Adobe Illustrator CS 6) of the sandpapers used ((**B**) rough, (**C**) medium, (**D**) fine) scaled identically to tooth scheme dimensions (**E**–**G**). (**E**–**G**) Schematic illustration (drawn with Adobe Illustrator CS 6) of tooth types (inner 15 marginals: stylus = yellow, cusps = orange; lateral I: basis and stylus = purple, cusp = light purple; lateral II: basis = green, thickening = dark green) of every second row (tooth rows 1–11), to depict area loss due to the sandpaper-tooth contact (wear patterns) for each sandpaper type (rough, medium, fine). (**H**) Degree of wear from posterior (slightly worn) to anterior (greatly worn).

**Figure 7:**
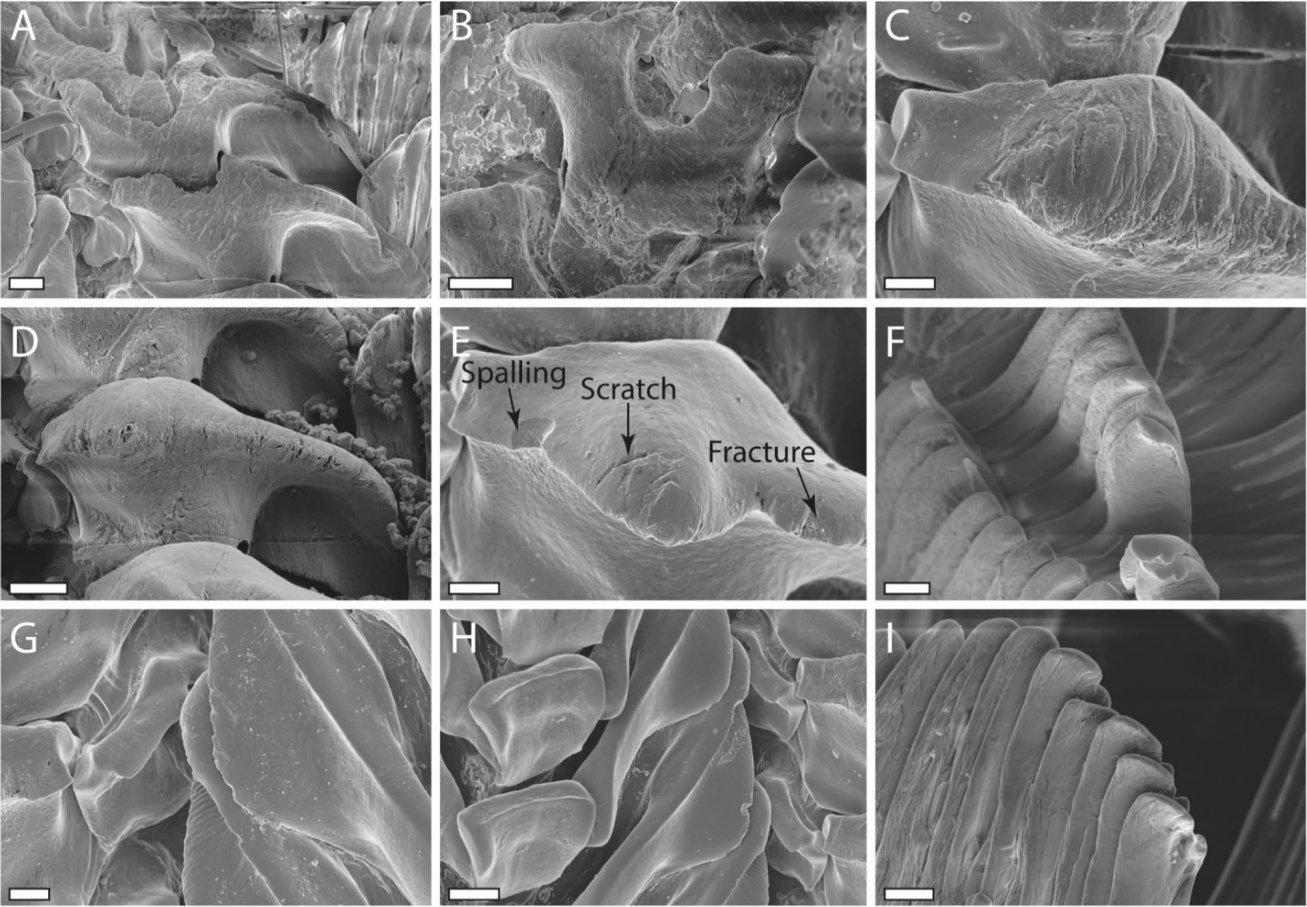
Detailed SEM images of worn radular teeth depicting different kinds of wear (spalling, scratch, fracture). (**A**–**E**) Lateral tooth II. (**F**, **I**) Inner marginal teeth. (**G**–**H**) Lateral tooth I. Scale bars: A, D, E, H = 20 μm, B, C, F, G = 10 μm, I = 5 μm.

Individuals were dissected, radulae extracted, food particles and tissues digested with proteinase K, according to the protocol of^39^, and cleaned by a short, ultrasonic bath. Radulae were arranged on scanning electron microscope (SEM) sample holders. To obtain images from all teeth wet marginal teeth were carefully stroked from central, lateral I, and lateral II. Then the radula was air dried, coated with gold–palladium, and documented with a Tabletop microscope TM 4000 Plus (Hitachi, Tokyo, Japan) or a Zeiss LEO 1525 (One Zeiss Drive, Thornwood, USA) (Figs. 1, 3, 4, 5, 6, 7).

Tooth rows of all specimens (N = 40) were counted and a median with standard deviation was calculated. For each radula, the width of two entire tooth rows from the working zone were measured. Their teeth were also measured to receive tooth width, length, height, and thickness (given as range). To receive the relative size of each tooth in percent (given as range in the results) we calculated the width of single tooth*100/the width of entire tooth row.

Wear patterns were identified by comparing teeth from specimens, which were fed on sandpaper substrate, with specimens from control group. So far no workflow could be established to document exact tooth volume loss precisely, because teeth are of low contrast and very small; thus a precise visualization by µ-CT technique is not possible. To however approach the topic of quantitatively analyzing radular wear we documented teeth by SEM. First, 2D images from above were used to approach area loss of tooth types, showing signs of wear (inner 15 marginal teeth, lateral tooth II, lateral tooth I); since slight wear started in row 11 we defined tooth area/volume of row 11 as 100%. Teeth of every second row were outlined, starting at row 11 receiving 6 outlined rows, with Adobe Illustrator CS 6 (Adobe Inc., San José, USA) and translated into areas (Fig. 6). Areas were transferred to Adobe Photoshop CS 6 (Adobe Inc., San José, USA), where number of pixels was read out for each area. By comparing areas of unworn teeth with areas of worn teeth, percentage of area loss was determined and could thus be quantitatively analyzed (Table 2). During the whole procedure we tried to document teeth from a similar angle which was enabled since SEM allowed rotation of samples. However, sometimes this was not possible. Then, either the radula was re-wetted with alcohol and mounted again on SEM stabs or these teeth could not be used for calculation of tooth area loss (please see Table 2 for *n* = quantity of area measurements used to receive mean values). Mean values of area loss were calculated employing JMP Pro, Version 13 (SAS Institute Inc., Cary, NC, 1989–2007). On teeth from the control group, fed on glass surface, we also identified some wear. However, the wear was so slight and not pronounced, that we defined the remaining tooth area and volume as 100%, because our methodology does not allow discriminating in such small ranges.

**Table 2:** Mean tooth areas of the tooth parts (cusp, stylus, basis) still present in every second row (tooth row 1 = outermost row [oldest and most frequently used one]; tooth row 11 = first [youngest] row with almost no signs of wear) after foraging on sandpaper (rough, medium, fine), in %. *n* = analyzed teeth for area loss calculation.

Tooth structure	Inner 15 marginal teeth						Lateral II									Lateral I					
	Cusp			Stylus			Cusp			Basis			**Stylus**			**Cusp**			**Basis**		
Sandpaper type	Rough	Medium	Fine	Rough	Medium	Fine	Rough	Medium	Fine	Rough	Medium	Fine	Rough	Medium	Fine	Rough	Medium	Fine	Rough	Medium	Fine
**Tooth row**																					
1	0.00	0.00	0.00	67.81	69.42	66.91	0.00	0.00	0.00	80.78	84.57	70.40	61.29	85.29	60.29	76.32	84.42	47.14	76.03	87.40	53.56
	*n* = 213	*n* = 201	*n* = 214	*n* = 211	*n* = 199	*n* = 216	*n* = 20	*n* = 20	*n* = 20	*n* = 19	*n* = 17	*n* = 16	*n* = 19	*n* = 17	*n* = 17	*n* = 19	*n* = 16	*n* = 18	*n* = 19	*n* = 16	*n* = 18
3	0.00	0.00	0.00	73.56	78.78	69.60	0.00	0.00	0.00	100.00	100.00	85.67	63.24	92.16	63.11	81.58	90.91	55.71	78.65	90.80	64.42
	*n* = 256	*n* = 255	*n* = 235	*n* = 257	*n* = 235	*n* = 215	*n* = 20	*n* = 20	*n* = 20	*n* = 16	*n* = 18	*n* = 15	*n* = 16	*n* = 15	*n* = 16	*n* = 17	*n* = 18	*n* = 16	*n* = 17	*n* = 18	*n* = 16
5	16.73	16.85	8.63	100.00	100.00	87.41	16.78	24.83	0.00	100.00	100.00	100.00	73.04	98.04	85.99	84.21	93.51	71.43	78.65	90.84	67.42
	*n* = 276	*n* = 234	*n* = 215	*n* = 272	*n* = 247	*n* = 217	*n* = 19	*n* = 17	*n* = 15	*n* = 19	*n* = 17	*n* = 15	*n* = 19	*n* = 17	*n* = 15	*n* = 19	*n* = 17	*n* = 19	*n* = 19	*n* = 17	*n* = 19
7	37.06	37.09	16.75	100.00	100.00	100.00	46.64	39.26	19.80	100.00	100.00	100.00	92.65	100.00	95.17	100.00	100.00	82.86	79.40	95.42	75.66
	*n* = 241	*n* = 267	*n* = 290	*n* = 239	*n* = 269	*n* = 265	*n* = 16	*n* = 19	*n* = 16	*n* = 16	*n* = 19	*n* = 16	*n* = 17	*n* = 19	*n* = 18	*n* = 17	*n* = 15	*n* = 16	*n* = 17	*n* = 15	*n* = 16
9	86.80	86.83	68.02	100.00	100.00	100.00	81.21	90.27	43.96	100.00	100.00	100.00	100.00	100.00	100.00	100.00	100.00	84.29	91.39	100.00	76.40
	*n* = 264	*n* = 294	*n* = 273	*n* = 272	*n* = 281	*n* = 267	*n* = 19	*n* = 19	*n* = 17	*n* = 20	*n* = 20	*n* = 20	*n* = 20	*n* = 20	*n* = 20	*n* = 19	*n* = 18	*n* = 15	*n* = 19	*n* = 18	*n* = 15
11	100.00	100.00	100.00	100.00	100.00	100.00	100.00	100.00	100.00	100.00	100.00	100.00	100.00	100.00	100.00	100.00	100.00	100.00	100.00	100.00	100.00
	*n* = 288	*n* = 291	*n* = 267	*n* = 288	*n* = 271	*n* = 265	*n* = 20	*n* = 20	*n* = 20	*n* = 20	*n* = 20	*n* = 20	*n* = 20	*n* = 20	*n* = 20	*n* = 18	*n* = 17	*n* = 16	*n* = 18	*n* = 17	*n* = 16

### 3D model and volume loss

**Figure 8:**
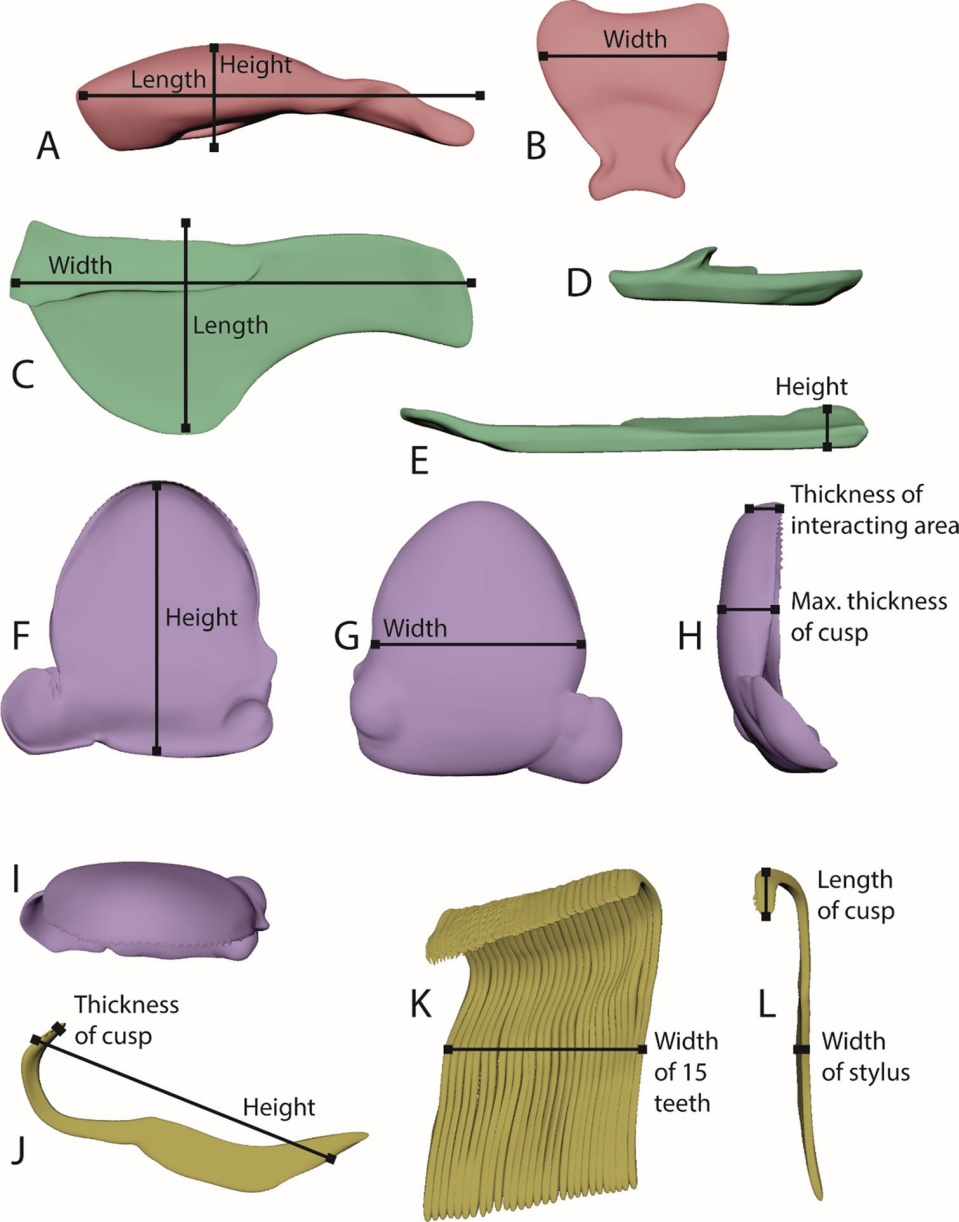
3D models of distinct tooth types for defining parameters measured (height, width, length, thickness). (**A**, **B**) Central tooth. (**A**) Lateral view. (**B**) Ventral view. (**C**–**E**) Lateral tooth I. (**C**) Ventral view. (**D**) Medial view. (**E**) View from anterior. (**F**–**I**) Lateral tooth II. (**F**) View from posterior. (**G**) View from anterior. (**H**) Lateral view. (**I**) Ventral view. (**J**–**L**) Inner marginal teeth. (**J**) Lateral view. (**K**–**L**) Ventral view.

**Figure 9:**
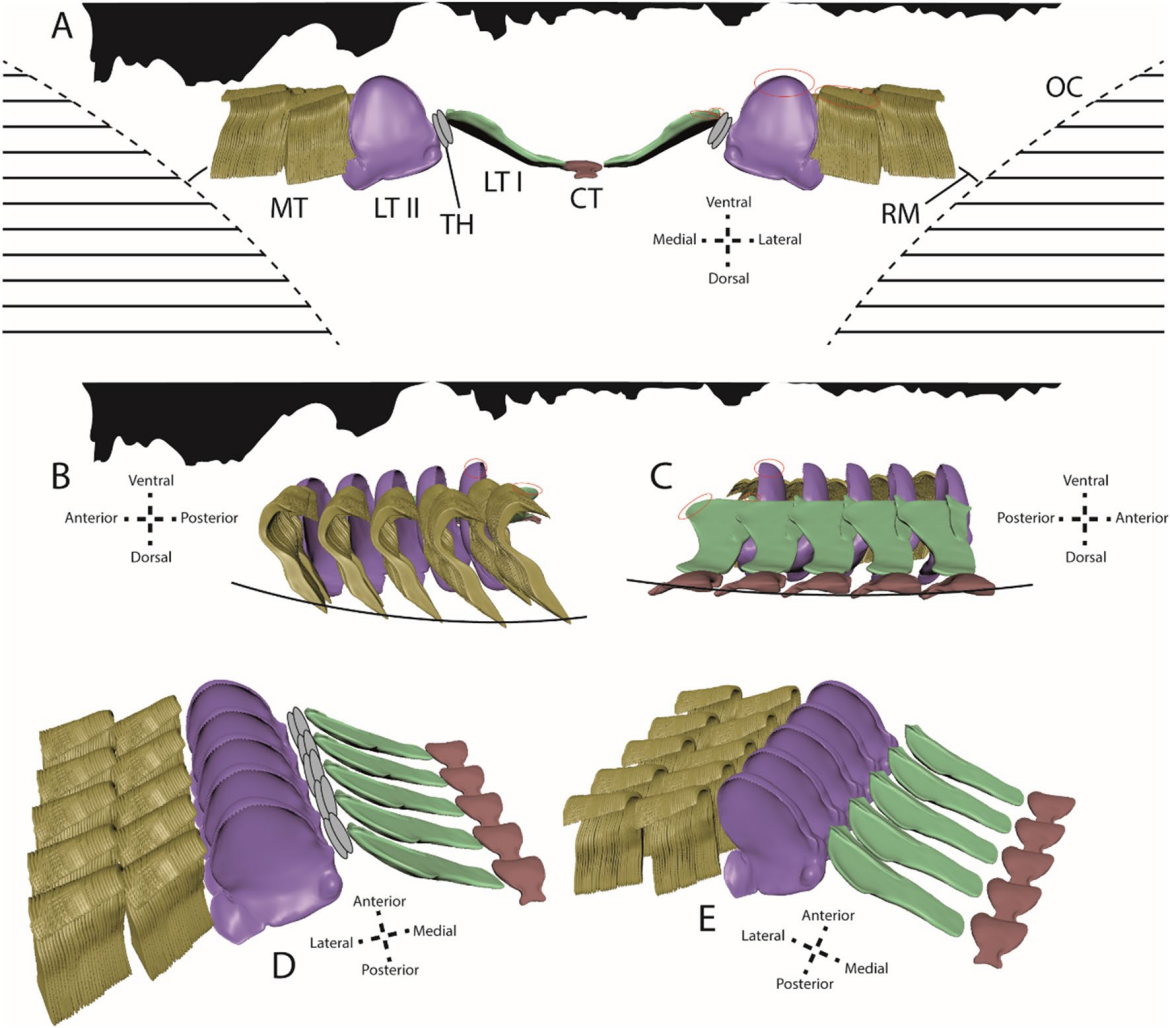
Visualization of proposed position of teeth on radular membrane during foraging, between the two odontophoral cartilages, from different views (see axes for anatomical orientation). Silhouettes (drawn with Adobe Illustrator CS 6) of sandpapers used (from left to right: rough, medium, fine) in (**A**–**C**) are scaled identically to dimensions of teeth. Contact areas are highlighted in red. (**A**) Frontal view depicting the inverse w-shape of membrane. (**B**–**C**) Lateral and medial views depicting additionally a curvature of the membrane towards dorsal. (**D**–**E**) Curvature from latero-ventral and medio-ventral view. CT = central tooth, LTI = lateral tooth I, LTII = lateral tooth II, MT = marginal teeth, OC = odontophoral cartilage, RM = radular membrane, TH = thickening of membrane/ interlocking teeth.

**Figure 10:**
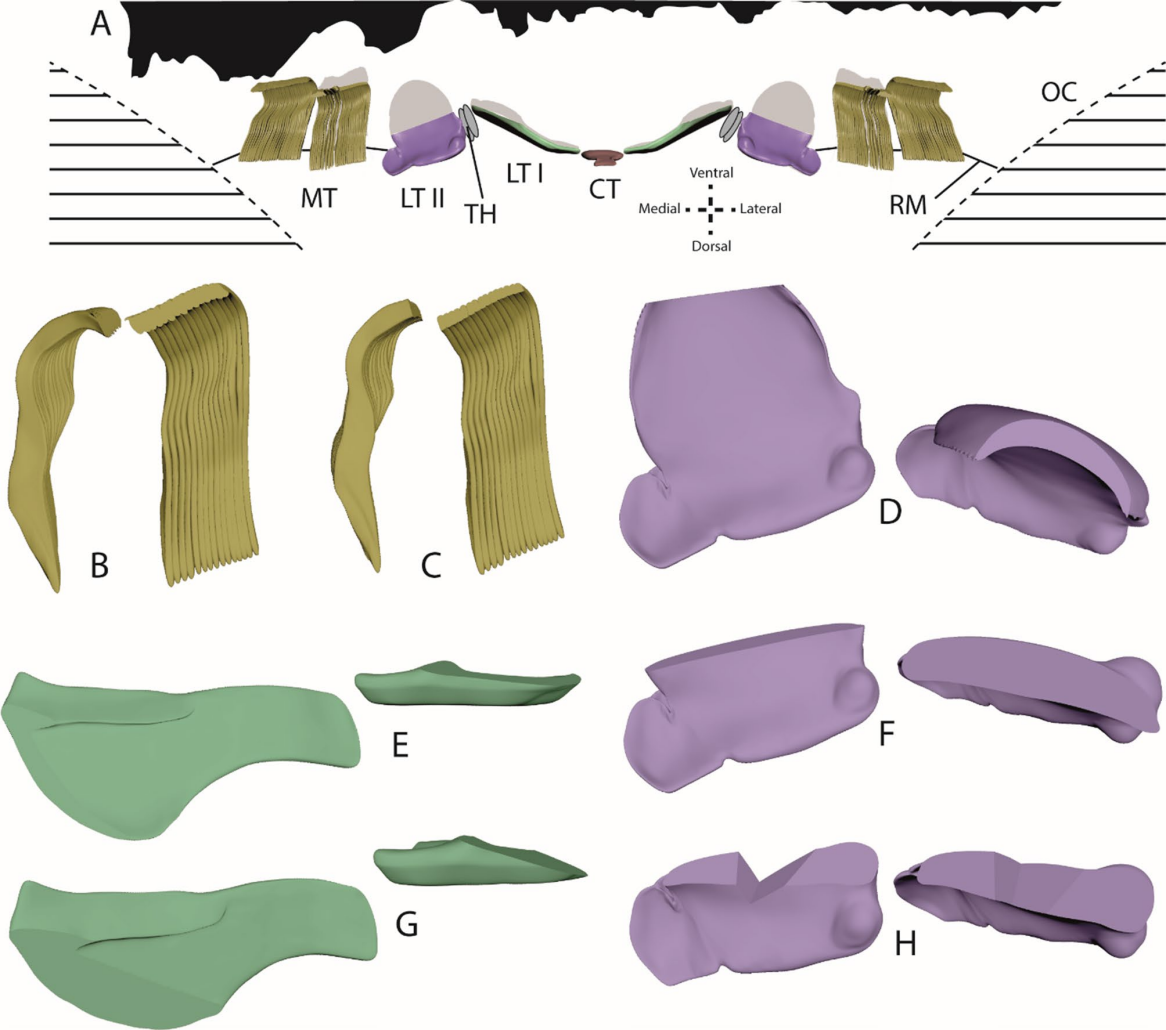
(**A**) Visualization of proposed position of the maximally worn teeth on radular membrane, between the two odontophoral cartilages, from frontal view (see axis for anatomical orientation). Silhouettes (drawn with Adobe Illustrator CS 6) of sandpapers used (from left to right: rough, medium, fine) are scaled identically to dimensions of teeth. In grey are silhouettes of unworn teeth. B–H. Some representable 3D models with worn condition (**B**–**C**) inner 15 marginal teeth, D, F, H. lateral tooth II, E, G. lateral tooth I). (**B**) Wear on 5th tooth row induced by rough sandpaper. (**C**) Wear on 1st tooth row induced by fine sandpaper. (**D**) Wear on 7th tooth row induced by medium sandpaper. (**E**) Wear on 1st tooth row induced by medium sandpaper. (**F**) Wear on 3rd tooth row induced by medium sandpaper. (**G**) Wear on 1st tooth row induced by fine sandpaper. (**H**) Wear on 1st tooth row induced by fine sandpaper. See Table 3 for volume loss in %.

To approach the topic of tooth volume loss, we created 3D models of teeth, which were also used for defining tooth sections and parameters as tooth width, length, etc. (Fig. 8), and for visualization of tooth position on radula membrane, including proposed shape during feeding (Figs. 9 and 10).

For this, teeth were formed manually, because they could not be visualized with µ-CT technique as it has been applied in previous studies of radular tooth morphology^10,40^. As described detailly in^41^, radulae of two additional specimens, sheltered without sandpaper, were extracted, digested, cleaned, and mounted on SEM sample holders. To obtain images from all sides of an individual tooth, the unworn but mature radular part was manually destroyed, teeth were extracted by tweezers, twisted, mounted, and finally visualized with Zeiss LEO 1525 SEM. Using 3D software Maya 2019 (Autodesk, Inc., San Rafael, USA), teeth were then virtually formed by hand, always comparing the computer model with the SEM images taken from different sides. To simulate the typically observed tooth wear patterns for each sandpaper types, which had been documented via SEM, the final 3D model of each individual tooth was altered by employing the tool ‘booleans: difference’ in Maya 2019 (Supplementary Fig. 1): manually modeled cubes or spheres were subtracted from the original tooth’s 3D model until the model (Fig. 10) looked similar to the worn teeth. Model volume before and after this procedure was calculated with Maya 2019 employing the tool ‘measurements’. Thus, tooth volume loss in % could be determined (Table 3).

**Table 3:** Calculated tooth volume of the teeth (lateral I, lateral II, inner 15 marginal teeth) present in every second row (tooth row 1 = outermost row [oldest and most frequently used one]; tooth row 11 = first [youngest] row with slight signs of wear) after foraging on sandpaper (rough, medium, fine), in %, estimated by manipulating the 3D model until it looks similar to worn teeth.

Tooth type	Inner 15 marginal teeth			Lateral II			Lateral I		
Sandpaper type	Rough	Medium	Fine	Rough	Medium	Fine	Rough	Medium	Fine
Tooth row									
1	73.11	73.95	72.69	37.53	39.81	36.59	80.58	83.06	76.86
3	78.15	78.15	73.11	37.73	45.11	36.59	87.60	91.32	78.10
5	80.25	80.25	78.15	67.78	70.69	47.40	93.80	95.45	78.93
7	92.86	92.86	80.25	83.06	81.19	67.67	100.00	100.00	83.47
9	97.90	97.90	96.22	100.00	100.00	82.95	100.00	100.00	87.19
11	100.00	100.00	100.00	100.00	100.00	100.00	100.00	100.00	100.00

### Feeding tracks

**Figure 11:**
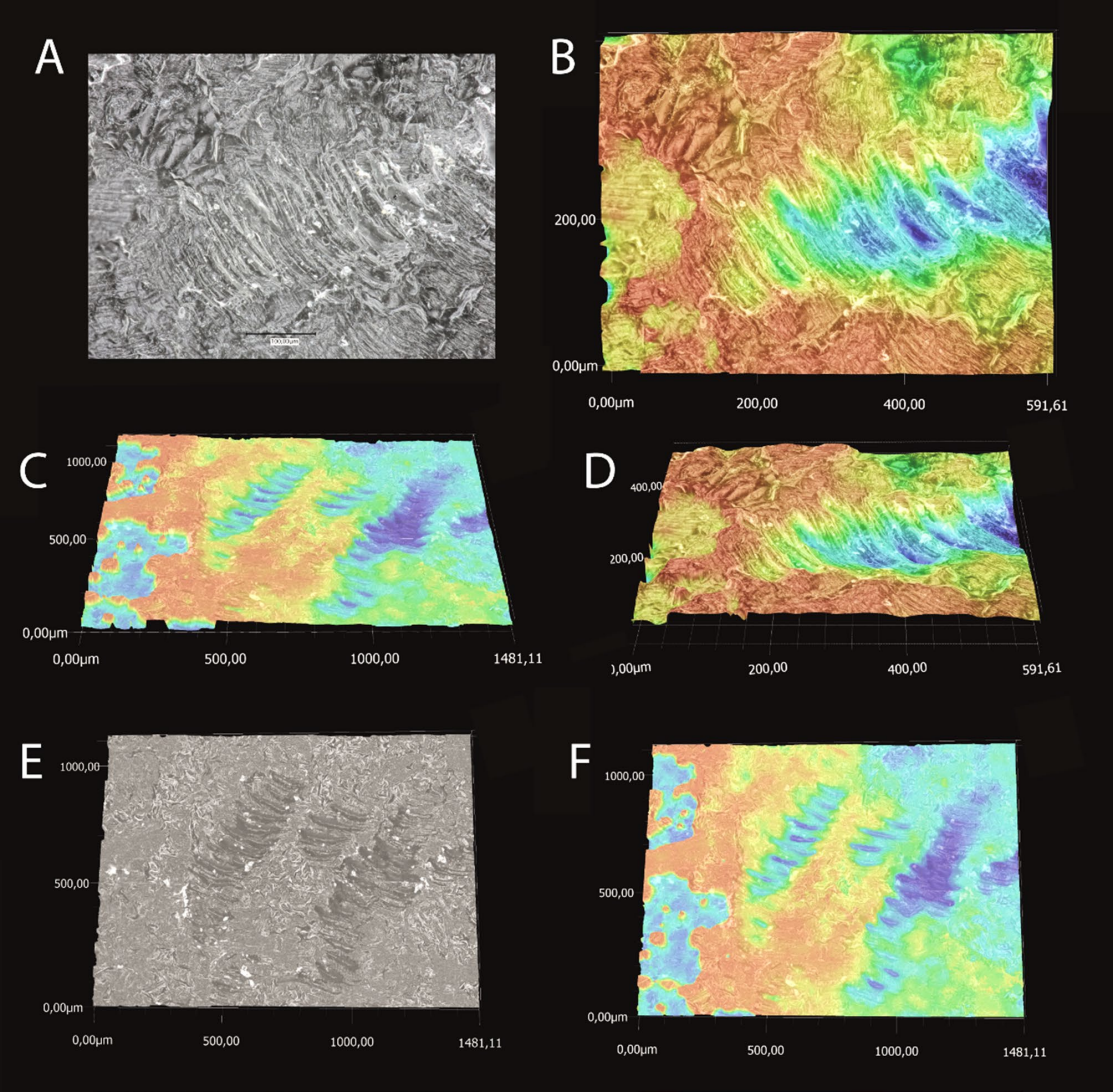
Feeding tracks left by gastropods on paraffin surface. Only the traces of lateral tooth II cusps are clearly visible. (**A**–**C**) Feeding track I from different perspectives: (**A**) Stacked image in SEM optic; (**B**, **D**) Colored surface texture visualized with Keyence VHX-7000. (**C**, **E**, **F**) Feeding track II from different perspectives: (**E**) Stacked image in SEM optic; (**C**, **F**) Colored surface texture visualized with Keyence VHX-7000. Dimensions are given in μm.

Feeding tracks were recorded by melting paraffin and applying it onto a petri-dish evenly. This was placed into one aquarium with 4 additional animals, which were not used previously in experiments. Algae paste was spread over it to encourage feeding on the surface. After one day, the petri-dish was dried, controlled by microscope, and most pronounced feeding tracks were visualized with a Keyence VHX-7000 (KEYENCE, Neu-Isenburg, Germany) (Fig. 11).

## Results

### Radular structure and feeding tracks

The radula of *V. turrita* possesses 150 ± 1 tooth rows along its longitudinal axis (for anatomical directions and radula from control group see Fig. 1). Each transversal row consists of one central tooth (or rachidian tooth, R), flanked to each side by one lateral tooth (lateral I), one large and dominant lateral tooth (lateral II), and approximately 40 thin marginal teeth. Thus, the following tooth row formula can be compiled: 40 + 1 + 1 + R + 1 + 1 + 40.

The central tooth is rectangular, 52–70 μm wide (6–7% of one transversal row), 51–74 μm long, and 40–43 μm high (all parameters measured are defined in Fig. 8). Lateral I exhibits a broad, shoulder blade-shaped basis and a 7–10 μm long thickening or edge. It is 135–173 μm wide (15–18% of one transversal row), at maximum 58–75 μm long, its medial part 28–33 μm high, and its lateral part 7–9 μm high. Lateral II is of roundish shape with a broad basis and stylus merging with a thick cusp, which possesses one larger and multiple smaller denticles; it is only 110–152 μm wide (14–15% of one transversal row), but dominant and prominent through its height (maximal 90–110 μm high). The central part of its cusp is thickened (33–39 μm), the part interacting with ingesta is thinner (9–17 μm thick). Between lateral I and lateral II, two smaller, interlocking structures are documented (e.g. Fig. 4E) which could be reduced teeth [see 42] or membrane hardenings. Both together are 42–50 μm wide (5–6% of one transversal row) and 52–77 μm high. Each marginal tooth consists of a stylus (8–12 μm wide) and a cusp. The inner 15 marginal teeth differ in their morphology from the outer 25 ones. The inner marginal teeth possess styli, which are 80–110 μm high and 20–30 μm thick, and cusps, which are 27–40 μm long and 3–4 μm thick; cusps contain few small denticles. The outer marginal teeth possess higher and thinner styli, which are 100–150 μm high and 3–6 μm thick, and shorter and thinner cusps, 18–27 μm long and 2–3 μm thick; they have more and thinner denticles. When all marginal teeth are manually fanned out and unfolded, they make up 43–45% of one transversal row. However, under natural conditions, these teeth rather cluster together and seem agglutinated. The radulae from control group exhibited almost no signs of wear. Only cusps of lateral tooth II leave feeding tracks on the paraffin surface (Fig. 11).

### Sandpaper-induced wear patterns

Wear is only found in outermost 11 tooth rows (7% of whole radula) and in lateral I, lateral II, and 15 inner marginal teeth (see Figs. 3, 4, 5 for wear; for schematic tooth area loss see Fig. 6). Central teeth as well as the remaining marginal teeth did not show signs of wear (Figs. 1, 3, 4, 5, 6).

Greatest wear (see Table 2 and Fig. 6 for tooth area loss; see Table 3 for tooth volume loss per tooth type and row) was documented in radulae from specimens foraging on the finest sandpaper (Fig. 3), followed by the roughest (Fig. 5), and finally the medium sandpaper (Fig. 4).

### The maximal wear at row 1 for whole row and whole teeth

The fine sandpaper (mean width of one abrasive particle equals 1–3% of one transversal tooth row width) causes a maximal transversal row area loss of 57% (loss measured in row 1) (see Table 2 and Fig. 6 for tooth area loss). Lateral I lost 50% of its area and 23% of its volume, lateral II 57% area and 63% volume, and the 15 inner marginal teeth together 67% area and 27% volume. The medium sandpaper (mean width of one abrasive particle equals 3–5% of one transversal tooth row width) leads to a maximal transversal row area loss of 41%. Lateral I loses maximal 15% area and 17% volume, lateral II 43% area and 60% volume, and the inner 15 marginal teeth 66% area and 26% volume. The rough sandpaper (mean width of one abrasive particle equals 15–23% of one transversal tooth row width) causes a maximal transversal row area loss of 48%. Lateral I loses maximal 24% area and 19% volume, lateral II 53% area and 60% volume, and the inner 15 marginal teeth 66% area and 27% volume.

### Wear on lateral tooth I

Greatest tooth area and tooth volume loss (see Table 2 and Fig. 6 for tooth area loss; see Table 3 for tooth volume loss per tooth type and row) were documented after interactions with fine sandpaper (one sandpaper particle equals 13–17% of tooth), followed by rough (one sandpaper particle equals 113–117% of tooth), and finally medium (one sandpaper particle equals 16–21% of tooth).

Great wear is documented on the lateral edges of the cusp, becoming thinner and thinner through ongoing interactions with the abrasive surface, and the lateral edges of tooth basis. By interaction with the fine sandpaper, the cusps lose an area of maximal 53% and the basis of 46% (measured in row 1) (see Table 2 and Fig. 6 for tooth area loss). For both tooth parts signs of wear appear in row 9. In the process of ongoing interaction between tooth and rough sandpaper, each basis loses maximal 24% area and each cusp 24% (measured in row 1). First signs of wear on basis can be found in row 9 and first signs of wear on cusp in row 5. After interaction with the medium sandpaper, lateral I was worn down least. Each cusp loses maximal 16% and each basis 13% of its area (measured in row 1). First signs of wear on cusp appear in row 9 and first signs of wear on basis in row 7.

### Wear on lateral tooth II

Lateral II shows overall greatest degree of wear and loss of both area and volume, starting at the medial part of its cusp (see Table 2 and Fig. 6 for tooth area loss; see Table 3 for tooth volume loss per tooth type and row). After the tooth cusp is worn down, stylus, and then basis is affected from wear, as they are exposed to the substrate.

Greatest tooth area and tooth volume loss was again documented after interactions with the fine sandpaper (one sandpaper particle equals 15–20% of tooth), followed by rough (one sandpaper particle equals 128–177% of tooth), and finally medium sandpaper (one sandpaper particle equals 18–26% of tooth) (see Table 2 and Fig. 6 for tooth area loss).

During interaction with fine sandpaper the ongoing wear, starting at the medial part of each cusp, leads to the complete loss of the cusp already in row 5. Thus, stylus and basis became exposed. With decreasing tooth cusp area, stylus interacts more frequently with abrasive substrate (first signs of wear were observed in row 7) until a u-like structure is retained at its maximal degree of wear (37% of stylus area loss in row 1). Wear on basis appears in row 3; 30% is lost in row 1. When foraging on rough sandpaper, the cusp is worn down completely already in row 3. Here, each stylus loses maximal 30% of its area (measured in row 1). The basis showed signs of wear only in row 1. Lateral II showed the smallest degree of wear after interaction with medium sandpaper. Here cusps are affected first at their lateral parts and completely lost in row 3; stylus (wear started in row 1; area loss of 15%, measured in row 1) and basis (wear started in row 1; area loss of 15%, measured in row 1) show very small signs of wear.

### Wear on 15 inner marginal teeth

Only the 15 inner marginal teeth showed signs of wear, appearing first at their cusps. Their styli always showed vertically running wear patterns; styli are worn down after cusps were lost. Medial teeth of inner 15 teeth were affected most and outer ones least.

Greatest tooth area and tooth volume loss was again documented after interactions with fine (one sandpaper particle equals 185–279% of tooth stylus), followed by rough (one sandpaper particle equals 1630–2246% of tooth stylus), and finally medium sandpaper (one sandpaper particle equals 234–351% of tooth stylus) (see Table 2 and Fig. 6 for tooth area loss; see Table 3 for tooth volume loss per tooth type and row).

Due to foraging on fine sandpaper, tooth cusps have already lost 32% of their area in row 9 (see Table 2 and Fig. 6 for tooth area loss). Additionally, styli and bases, especially of medial teeth, showed heavy signs of wear. In row 5, cusps possessed only half of the area than cusps in same row from individuals fed on rough and medium sandpapers. After interaction with rough sandpaper, first signs of wear on cusps were found in row 9. First, denticles become eroded, then cusps are lost (medial marginal teeth have lost their cusp in row 3), and teeth lose their height. After loss of cusps, bases become exposed and are affected by wear (medial marginal teeth showed wear at their basis in row 5; tooth area loss 32%, measured in row 1). The medium sandpaper induced wear patterns at tooth basis and cusp (medial marginal teeth showed wear in their basis in row 5; tooth area loss 31%, measured in row 1) are similar to patterns induced by fine sandpaper. The only differences were observed for the bases; they were less worn down by medium sandpaper.

### Appearance and development of wear

Unnatural wear can appear in three distinct forms: scratches, fractures, and spalling (Fig. 7). Feeding on a glass surface does not cause such impacts on the radula of *V. turrita*. First interactions with abrasive substrate asperities usually leads to well-defined scratches on the tooth surfaces. With ongoing interaction with sandpaper, fractures in tooth material can be detected, subsequently followed by spalling.

## Discussion

### Position of teeth on membrane and contact-areas

The position of teeth on the radular membrane while foraging is essential for understanding component’s functionality as each component may contribute differently to stress distribution or to prevention of structural failure, depending on its angle during contact with substrate and on its interactions with other teeth. In many previous works, it was assumed that the radula is pulled parallel and flat across the odontophoral cartilages by muscles and that teeth are oriented thus more or less parallel to each, other, presumably functioning like a rasp, a pulley, or a conveyer belt^11,19,43–47^. This configuration would be more or less similar to radulae mounted on SEM stubs (Figs. 1, 3, 4, 5, 6). However, by manually protruding the radula from dead gastropods, it was documented that the radula is not flat outside the mouth, but it of rather curved shape, with teeth oriented in semi-circles and distinct angels to one another^48^. Unfortunately, this hypothesis again does not reflect the exact configuration of teeth during feeding, since the radula in^48^ was pulled quite far outside the mouth opening. By observing feeding gastropods through transparent glass, complex motion patterns, highly distinct between taxa, and a bent shape of radula can be documented (for *Vittina* see^16^; for other gastropods see^11–15^). However, this approach does not precisely allow identification of a) position of teeth relatively to each other, and b) precise contact areas of teeth with the surface, due to the smallness of the radular teeth. Analyses of radular feeding tracks on flat surfaces provided important indications that teeth are capable of moving, twisting, and rotating during foraging^2,11,12,17,23,48^ but again, precise tooth-ingesta-contact areas are hard to determine. The here presented experimental approach, involving sandpapers with distinct dimensions of asperities and the analysis of sandpaper-induced wear facets, allowed the determination of radular parts, interacting with substrate, as it had been done for stylommatophoran gastropods^37^. Furthermore, inferences about the relative position of teeth on the radular membrane during foraging can be deduced from the ontogeny of wear.

### Ontogeny of wear and bent shape

First wear traces appear on the medial part and the large denticle of lateral II cusp. Once denticle height decreases, both lateral edges of lateral I interact with the substrate and become worn later. The 15 inner marginal teeth also show signs of wear at their styli and cusps after the lateral II cusps are affected and worn down. Here, a gradually increasing wear from medial to lateral side within the array of marginal teeth is observed. The central tooth, the medial part of lateral I, and the outer marginal teeth—even though they are the longest radular teeth—do not show any signs of wear, which in turn means that they all do not directly interact with the substrate while foraging. That not all teeth are used for foraging had also been shown for Stylommatophora^37^; in these experiments we found that with increasing substrate roughness more and more teeth interact with the surface. For *Vittina*, however, we observed that with increasing surface roughness the quantity of teeth involved in foraging does not change, only the degree of wear. This observation leads to the hypothesis that the radular membrane is bent during feeding, probably in the shape of an inverse w along the transversal axis (Figs. 9A, 10A). Additionally, wear on lateral I appears first on the lateral, posterior edge of its basis. Later wear appears on the large, anterior ‘cutting’ edge, even though this edge is higher than the lateral edge of the basis. Thus, the radula is additionally bent along its longitudinal axis during foraging (Fig. 9B–E).

Such bending behavior had been previously described for ‘Archaeogastropoda’, that bent and unfold their radula to build a broad area for grazing activity, followed by a folding in opposite direction to obtain a smaller structure, which can be stored in the head^49^. Neritidae seem to bend their radulae in such a way, that the lateral teeth II are closer to the substrate than the medial ones^16,37^. Here, not the center of the radula, but rather its lateral areas will be mechanically stressed^42^.

### Potential origins of bent shape

The radula is situated between two large odontophoral cartilages (Figs. 9A, 10A)^50–52^ which are controlled by buccal mass musculature. They could establish a lateral force, possibly supported by the mechanical properties of the radular membrane, leading to a bent shape in *V. turrita*. Each central tooth together with the adjacent two lateral teeth I probably span the radula along its longitudinal axis. Additionally, the two membrane thickenings or reduced teeth [see 42] act like joints, probably stabilizing the entire structure. Thus, radular teeth are not oriented parallel, either to each other or to the odontophoral cartilages, during foraging, which had also been observed for docoglossan radulae of Fissurellidae^10^.

### Unnatural wear patterns and prevention of failure

Our here presented documentation of wear patterns is not as precise as it had been conducted for e.g. vertebrate teeth; we however think that it is a step towards a quantitative analysis of wear. We found that sandpaper has a high impact on tooth wear. Largest abrasion and deepest scratches were induced by feeding on fine sandpaper (Figs. 3, 6). Radulae from specimens sheltered on rough and medium sandpapers show a smaller degree of abrasion (Fig. 6, Tables 2, 3), even though the abrasive particles have a larger diameter (Fig. 2, Table 1). In the case of the rough sandpaper, the particles are even larger than the teeth, and we expected higher impacts from foraging on the larger particles than on the smaller ones. Additionally, scratches on teeth from specimens sheltered on fine sandpaper are fine and deep, presumably induced from single, sharp-edged abrasive particles, indicating that teeth do rather not bend or swerve. Worn teeth from gastropods, sheltered on both rougher sandpapers, rather show fractures and spalling, which indicates that teeth seem to be cushioned to a certain extent. Either a flexible tooth embedment in radular membrane allows the spreading of teeth to the sides^37,41,53,54^ and/or a softer cushioning beneath the membrane, the odontophoral cartilages, allows the swerving in dorsal direction when teeth hit larger asperities^4,6,37,55–58^. Similar mechanisms have been observed for the anchorage of mammalian teeth^59,60^.

Natural wear has been documented in various molluscan species^6,8,10,24,46,61–67^, but wear-causing agents have not been reported in these publications. Comparing results from previous studies with the ones obtained here depict that the natural wear in Polyplacophora and Patellogastropoda^24^ is heavier than the wear observed in the radulae from our control group. This could be an artefact from the experiments as our control animals were sheltered without any abrasiveness. To determine if the natural wear in *Vittina* is also as small, gastropods should be collected directly in natural habitat. Polyplacophora and Patellogastropoda loosen thick and dense algae covers from solid substrate in the surf zone^68^. *V. turrita* also inhabits solid substrates, but seems to prefer rather porous ingesta^37^. In the first case, the molluscs probably have to exert higher forces to loosen ingesta, presumably resulting in heavy wear, whereas in the second case gastropods probably have to exert smaller forces, resulting in less wear. Additionally, docoglossan teeth are harder and stiffer resulting from iron incorporations^69–71^ whereas neritid teeth are rather soft and more flexible, as they are rather chitinous^72^. Softer teeth probably allow more elastic deformation without damage when hitting a large obstacle, leading to less wear or structural failure^54^. Sandpaper-induced wear patterns in *V. turrita* are more pronounced than any natural wear documented (for references see above) indicating the limitations of this feeding structure.

### Tooth function

The functionality of a grinding tool is—among other parameters—highly determined by its contact areas, which are again determined by the tool’s morphology^34^. Contact areas between the radula of *V. turrita* and the ingesta surface are, as stated above, the lateral edges of lateral I, the medial edge of lateral II, and the cusps and the styli of the 15 inner marginal teeth. If a tool/tooth is rather pointy, its contact area is smaller, which in turn means that more pressure can be exerted on the target surface during ‘puncturing’ (^73^ for radular teeth;^34^ for review on ‘puncturing’). Thus, often a small contact area indicates that this part of a structure is rather used for piercing. We think, that the small and pointy denticles on the lateral II cusps could be used for piercing in *V. turrita*. When the denticles are worn down, the gastropod forages with the remaining lateral II cusp, which seems to be the main tool for ingesta loosening^74^. This is also inferred from the analysis of feeding tracks (Fig. 11) as only tracks from lateral tooth II were found. Lateral I does not possess any denticles, but rather two large edges on the lateral side, which interact with the substrate, possibly cutting off ingesta. The 15 inner marginal teeth have contact areas to the substrate at their cusps and styli, proven by wear on both parts. However, marginal teeth are probably not used for puncturing, since they are flexible. They rather brush across the substrate^74^ and collect particles with their denticles (for the relationship between hardness and capability of scratching)^15,41,54,73,75^. The hypothesis that neritinomorph marginal teeth are rather used for collecting particles due to their softness and flexibility, whereas both lateral teeth are rather used for loosening ingesta from surfaces had been put forward previously^46,74,76^ and is well supported by the result of the here present study. However, as the inner marginal teeth show also signs of wear, they are probably, at least temporarily, involved in more intense tooth-ingesta interaction, also potentially loosening ingesta. This is well supported by analyses of their morphology: the inner marginal teeth possess a thicker and shorter stylus whereas the outer marginal teeth are rather thin and slender. Thus, the inner marginal teeth are potentially rather capable of exerting higher forces than the outer marginal teeth (for the relationship between radular tooth morphology and function see also^41,54,73,75^). Chemical analyses of the radula of the neritid *Nerita atramentosa*, documenting that the medial marginal teeth contain more minerals than the outer ones^72^, additionally support this hypothesis. However, whether this is also the case for *V. turrita* awaits further investigation.

The stylus and the basis of each tooth probably distribute mechanical stress from their interacting parts to the radular membrane during foraging. Additionally, teeth stabilize each other laterally, leading to stress distribution from the medial to the lateral side of the radula^46,73,76^.

Previous hypotheses for neritid radulae, stating that the central teeth and the medial edges of lateral I are used for scratching across the ingesta surface loosening particles^46,74,76^, could not be supported by our analyses. Our results clearly show that both structures do not interact with substrate. Since the central tooth interacts with the adjacent lateral teeth I through joint-like edges (for tooth-tooth contact in neritids^46,76^), these teeth together probably span the radula to its bent shape. The fact that not all radular teeth are involved in the loosening of ingesta (e.g. central teeth) had also been documented for Fissurellidae^10^.

### Self-sharpening

Wear patterns on all tooth edges are rather curved and roundish. After the cusps break off, e.g. in lateral tooth II, gastropods seem to forage with the remaining stylus, until this structure is also worn down [see also 46]. We did not find any signs of a self-sharpening effect as previously described for chitons, limpets, and echinoderms^10,24,65,77,78^. However, obviously damaged teeth can, to a certain extent, still maintain their function.

## Conclusion

By this experimental approach we identified contact areas between *Vittina* radula and the sandpaper substrate and were thus able to build hypotheses about tooth function. From contact areas and ontogeny of wear we could also infer the radular configuration during foraging, which is of bent shape along two axes. This shows that a deduction about tooth function from analyzing mounted radulae is problematic, as their configuration is manipulated and does not resemble the feeding configuration. Our results additionally contribute to the topic of structural failure prevention in radulae.

## Supplementary Information

Supplementary Information.
