# Supplementary material for: Feeding experiments on Vittina turrita (Mollusca, Gastropoda, Neritidae) reveal tooth contact areas and bent radular shape during foraging
Source: Sci Rep. 2021 May 5;11:9556. doi: 10.1038/s41598-021-88953-7 (PMC8099886; doi:10.1038/s41598-021-88953-7)
Supplement: Supplementary file 1 — Supplementary Information. [file 41598_2021_88953_MOESM1_ESM.pdf]

Feeding experiments on *Vittina turrita* (Mollusca, Gastropoda, Neritidae) reveal tooth contact areas and bent radular shape during foraging

WENCKE KRINGS, CHRISTINE HEMPEL, LISA SIEMERS, MARCO T. NEIBER, STANISLAV N. GORB

Supplementary:

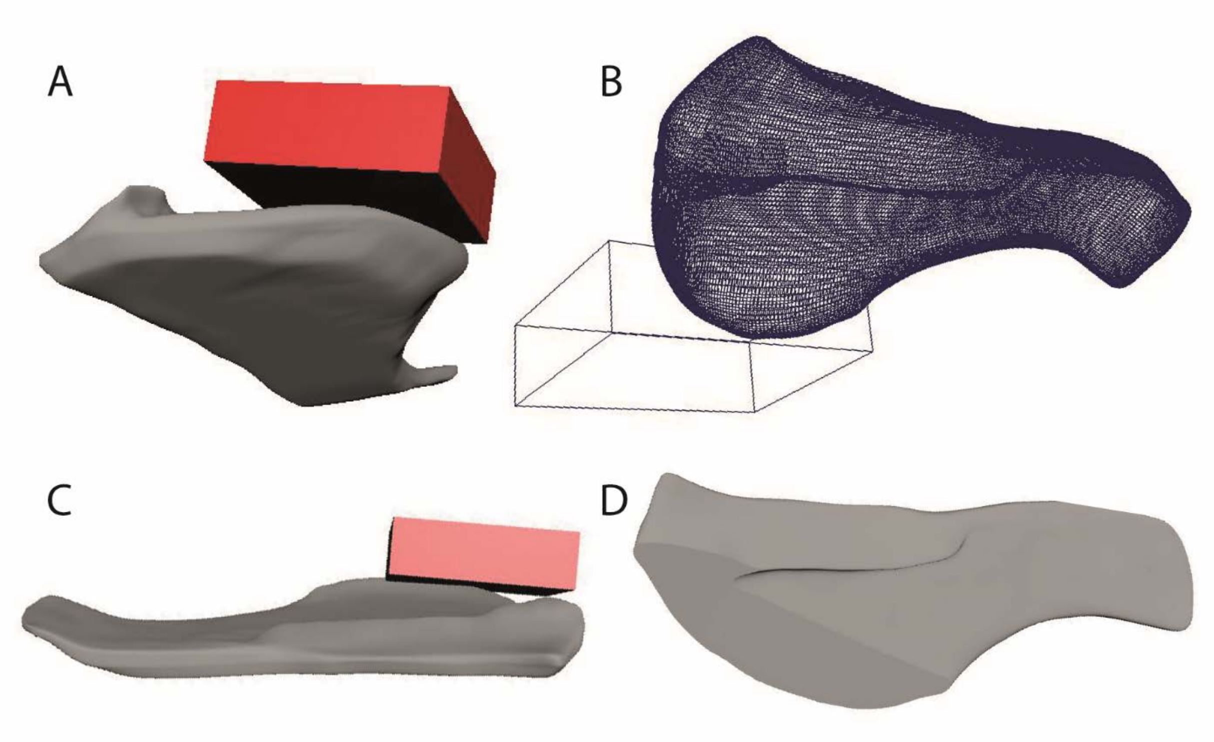

Supplementary Fig. 1. To simulate the typically observed tooth wear patterns the final 3D model of the individual tooth type (here the lateral tooth I) was altered by employing the tool 'booleans: difference' in Maya 2019. Manually modeled cubes (red-colored in A,C, with transparent faces in D) or spheres were subtracted from the original tooth's 3D model until the remaining model (D) looked similar to the worn teeth.
